# An Evolutionary Computation Approach for Optimizing Multilevel Data to Predict Patient Outcomes

**DOI:** 10.1155/2018/7174803

**Published:** 2018-03-18

**Authors:** Sean Barnes, Suchi Saria, Scott Levin

**Affiliations:** ^1^Department of Decision, Operations & Information Technologies, Robert H. Smith School of Business, University of Maryland, College Park, MD, USA; ^2^Department of Computer Science, Johns Hopkins University, Baltimore, MD, USA; ^3^Department of Emergency Medicine, Department of Civil Engineering, Johns Hopkins University, Baltimore, MD, USA

## Abstract

Widespread adoption of electronic health records (EHR) and objectives for meaningful use have increased opportunities for data-driven predictive applications in healthcare. These decision support applications are often fueled by large-scale, heterogeneous, and multilevel (i.e., defined at hierarchical levels of specificity) patient data that challenge the development of predictive models. Our objective is to develop and evaluate an approach for optimally specifying multilevel patient data for prediction problems. We present a general evolutionary computational framework to optimally specify multilevel data to predict individual patient outcomes. We evaluate this method for both flattening (single level) and retaining the hierarchical predictor structure (multiple levels) using data collected to predict critical outcomes for emergency department patients across five populations. We find that the performance of both the flattened and hierarchical predictor structures in predicting critical outcomes for emergency department patients improve upon the baseline models for which only a single level of predictor—either more general or more specific—is used (*p* < 0.001). Our framework for optimizing the specificity of multilevel data improves upon more traditional single-level predictor structures and can readily be adapted to similar problems in healthcare and other domains.

## 1. Introduction

Rapid accumulation of electronic health record (EHR) data and emphasis on meaningful use of health information technology (HIT) [1] has given rise to many modeling applications that attempt to predict individual patient outcomes. The majority of these prognostic models target clinical outcomes (e.g., mortality, acute myocardial infarction, and septic shock); however, others aim at predicting service-oriented outcomes that span operations (e.g., wait times and length of stay), cost, quality, and patient satisfaction [2–10]. Regardless of outcome, these models aim at improving healthcare delivery by supporting provider and organizational decision-making.

EHRs are a valuable source of input data commonly leveraged for these predictive applications. However, the heterogeneity, large-scale nature, and variability in data entry create challenges with respect to how to optimally specify these data for predictive models. Multilevel data describing patients' clinical conditions and medical interventions are commonly hypothesized predictors available in EHRs, but present unique challenges for model specification.

Multilevel data describes individual patient characteristics at multiple levels of specificity (see Table 1). For example, the International Classification of Diseases (e.g., 9th Revision, Clinical Modification or ICD-9-CM) contains more than 14,000 diagnosis codes and 3900 procedure codes used to classify the conditions of patients and the services they receive [11]. Diagnoses and procedure codes have inherent hierarchical structure represented by digits and decimals. For example, ICD-9-CM code 038.12 may be deconstructed from the lowest-to-highest level of specificity in the following manner:

- Level 1: 001–139 infectious and parasitic diseases
- Level 2: 030–041 other bacterial diseases
- Level 3: 038 septicemia
- Level 4: 038.1 staphylococcal septicemia
- Level 5: 038.12 methicillin-resistant *Staphylococcus aureus* septicemia

Tools such as the U.S. Agency for Healthcare Research and Quality's Clinical Classifications Software (CCS) may similarly introduce their own conceptual structure [12]. Documentation of medical history and chronic conditions is also defined by a multilevel structure (see Table 1), for example, “diabetes” (low specificity) or type I, type II, or gestational diabetes (high specificity). Medications provide additional examples, for which definitions can be more general classes (e.g., antibiotics), more specific subclasses (e.g., penicillin), or somewhere in between (e.g., broad versus narrow spectrum).

Hypotheses may be generated about the level of specificity needed to best differentiate patients with respect to the outcome predicted. However, often, it is unclear which level will be most effective. Further, the optimal level of specificity may change for different outcomes or even the same outcome in different populations. For example, there is a substantial body of work involving the prediction of readmission for patients who undergo coronary artery bypass graft (CABG) surgery [13–16]. In much of this work, there are risk factors for comorbidities, medications, and complications that could be defined more generally or more specifically, and minimal rationale was provided about how these modeling decisions affected model performance. In addition, the optimal levels of specification for these risk factors for predicting readmission rates for CABG patients may not translate to predicting a different outcome such as mortality.

In many cases, the specification of multilevel data is hypothesis driven, in that an initial judgment on the appropriate level of specificity is made and that specification is retained throughout the modeling process. We propose a framework for learning the appropriate level(s) of specificity from data, and we evaluate the trade-offs of flattening or retaining the hierarchical structure of these multilevel predictor data. In the first case (i.e., flattening), general and specific categories are collapsed into a single mutually exclusive level. Patients are initially placed in their most general category, and then patients with indications for more specific categories are extracted from their general categories. In this case, there is a fundamental change in the structure of the multilevel data, as patients with the same general category are now distinct from one another (i.e., some patients in the general category will retain that category, while others will convert to a more specific category). This redefinition differentiates this problem from a simple feature selection problem, whereby categories that contribute to the predictive performance (with respect to the desired outcome) are retained and others are excluded. In the case for which the hierarchical structure is retained, patients with indications for more specific categories will also retain indications for their general category.

There has been previous research focused on modeling with multilevel data structures, particularly in the areas of political science, psychology, sociology, public health, and education [17–23]. In this work, the notion of multilevel data relates to predictors that are collected at multiple hierarchical levels, for example, at individual and group levels (e.g., class, school, department, organization, and district). For example, Burstein [22] proposes a structure in which background, educational process, and outcome variables are measured at the individual (i.e., student) and group (e.g., community, school, and district) levels. Similar types of research exists in the healthcare space, with much of it falling within the health service research subfield [24–28]. For example, Sjetne et al. [28] developed a model to explain the variation in patient satisfaction (measured as percentage ratings across 10 categories) as a function of both individual patient (e.g., age, gender, education level, and length of stay) and hospital (size and teaching status) characteristics. The bulk of the existing research on multilevel data follows this approach and is inherently different from the problem that we present. In our approach, we focus only on data specified at the individual (patient) level, albeit at varying levels of specificity.

In the computer science field, there have been some recent works that are more closely related [29, 30]. Schulam and Saria [29] developed a learning framework to predict clinical trajectories using information measured at multiple levels of specificity (i.e., population, subpopulation, and individual). This general approach is similar to the aforementioned research, but the key difference is that their proposed method learns the relative importance of each level of the hierarchical structure, based on its ability to predict the desired outcome. In Choi et al. [30], the authors develop a graph-based attention model (GRAM) that leverages an existing hierarchical system (such as ICD or CCS) to predict diagnosis and heart failure outcomes. The attention mechanism primarily balanced the need for specificity of information with the observed sample size of that predictor in the training data. This approach was designed to address a specific limitation of deep learning models (in healthcare) that typically lack the requisite sample size for accurate training. Overall, our objective is similar in that we develop a learning framework for adapting hierarchical data structures for individual patient predictions, and this previous work underscores the need to develop such methods. However, we believe that our approach is more easily applied and more flexible and preserves the hierarchical predictors for interpretation by practitioners.

In the next section of this article, we define the general evolutionary computation (EC) framework. Then, we demonstrate the performance of this approach in predicting critical outcomes for emergency patients across five patient populations. After that, we discuss the implications of this approach and how it can be applied more broadly. Lastly, we conclude with some final thoughts and some proposals for future development.

## 2. Methods

We present a general EC framework for optimizing multilevel data for predictive modeling. This framework is suitable for both classification and regression problems. First, we introduce the reader to a case study of predicting critical outcomes for emergency department patients, which provides a specific context for which to present the framework. Then, we describe the framework itself, which can be readily adapted to other applications within healthcare and other domains.

### 2.1. Case Study: Predicting Critical Outcomes for Emergency Department Patients

Emergency Departments (EDs) have experienced a surge of patient volume to over 136 million visits annually in the United States (US) [31]. This has exacerbated the ED crowding crisis and places patients at undue risk of adverse events associated with delays in care [32, 33]. EDs are required to see all comers, thus patients must be quickly evaluated at presentation to determine the urgency of care needs. This process is called triage and has standards in place that require the provider to record the patient's demographics (age, gender), elicit a chief complaint (i.e., reason for visit), and measure vital signs (heart rate, respiratory rate, temperature, blood pressure, and oxygen saturation). Triage standards in the US require clinicians to apply the Emergency Severity Index (ESI), an algorithm used to assign patients to a 5-level scale from 1 (high severity; need for immediate treatment) to 5 (low severity; nonurgent) [34]. ESI relies heavily on provider judgment, is subject to high variation [35], and poorly differentiates a large majority group (ESI level 3), counter to the true objective of the triage [36, 37].

Thus, an alternative, outcome-based approach for conducting triage has been developed and is being used in several EDs in the US [37, 38]. A key component of this data-driven approach involves predicting critical care events for ED patients based on the information collected at presentation. Here, we define a critical care event as a composite and binary outcome that includes in-hospital mortality, direct admission to a hospital intensive care unit, or emergent surgery or catheterization for the same patient stay. These outcomes are analogous to the types of outcomes that would require immediate action on the part of care providers when the patient arrives in the ED and correspond to the most urgent ESI levels (i.e., one and two). This critical care event is the outcome that we aim to predict with our model.

In this study, we apply our EC framework to optimize multilevel predictors—specifically chief complaints—for predicting critical care events for ED patients. This prediction model utilizes the same information that is collected for the traditional triage process and includes the age, gender, and arrival mode of the patient, along with the aforementioned vital signs and the chief complaints that will be optimized using our EC framework. The vital signs were (nonuniformly) discretized into clinically meaningful categories, including a dedicated category for missing information [37, 38]. We summarize the categorical predictor variables in Table 2.

We apply our method across five patient populations, including a large, urban academic medical center (ACAD), a medium-sized community hospital (COMM), international hospitals in Brazil (BRAZIL) and the United Arab Emirates (UAE), and the nationally representative National Hospital Ambulatory Medical Care Survey (NAT). We provide summary characteristics of these five patient populations in Table 3.

### 2.2. General Evolutionary Computation Framework

Evolutionary computation is a class of metaheuristic algorithms that mimic biological processes to solve difficult optimization problems [39]. Relative to exact algorithms, evolutionary algorithms are stochastic and are not guaranteed to find global optima; however, they work well in practice and can provide good solutions within manageable computation times. In addition, evolutionary algorithms provide a flexible framework that can be readily adapted to different types of problems or variations of similar problems.

Specifically, we utilize a genetic algorithm (GA) to search for the optimal combination of complaints and complaint categories, for which the complaints represent more specific information on each patient's reason for visit and the complaint categories combine specific complaints into clinically meaningful groups. GAs imitate the process of natural selection, whereby stronger candidate solutions survive and weaker candidate solutions are eliminated [40]. We implemented our GA using the distributed evolutionary algorithms in Python package [41].

**Figure 1:**
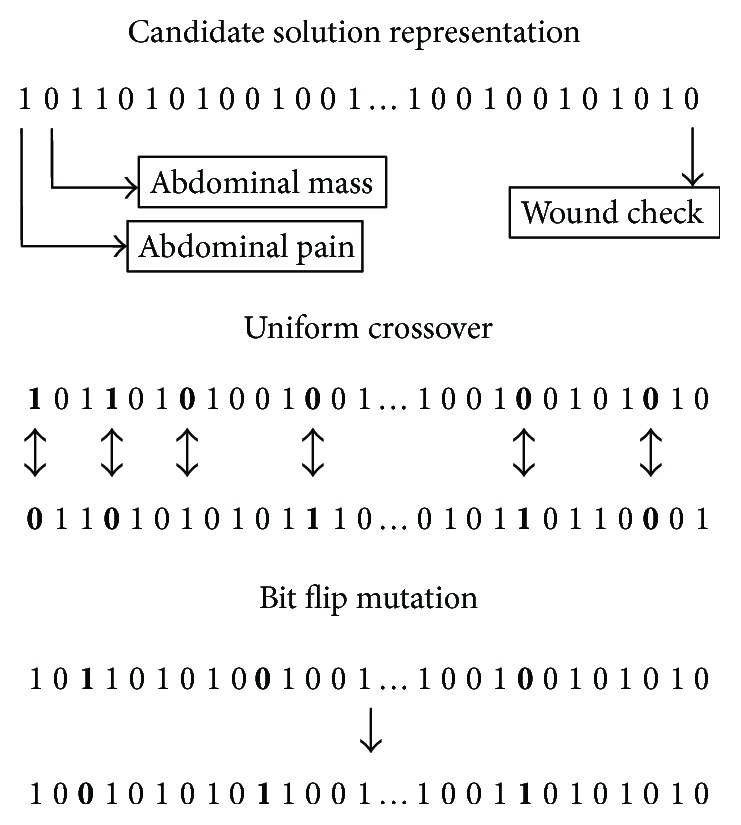
Genetic algorithm representation and recombination operators.

For this application of a GA, candidate solutions in the population are represented by binary bit strings of length *n*—where *n* represents the number of specific complaints—for which each bit *b_i_* represents whether a specific complaint is excluded (0) or selected (1) as a predictor in the classification model for the critical care outcome in ED patients. We include all aforementioned age, gender, arrival mode, and complaint categories in the prediction model and therefore do not need to include them in the search process. The population contains *N* candidate solutions, each of which is initialized with randomly generated 0 and 1 values (i.e., a random selection of specific complaints). For each generation, a subset of the population is selected via a tournament selection scheme for crossover operations. Uniform crossover is ideal for this application (as opposed to other common crossover operations such as single- or multipoint crossover) because there is minimal advantage in preserving contiguous blocks of chromosomes (i.e., each selected complaint is essentially independent from the others). Once crossover is completed, a subset of candidate solutions in the new generation is selected for mutation. We utilize a simple bit flip operation for mutation, which inverts a subset of complaint bits within each candidate solution. For example, complaints selected for mutation that are currently excluded become selected, and complaints selected for mutation that are currently selected become excluded. We summarize the representation of candidate solutions and the crossover and mutation operations in Figure 1. Control parameters for the GA (summarized in Table 4) were selected via experimentation to maintain the diversity of the population and prevent premature convergence toward a suboptimal solution. In general, there is evidence that a broad range of control parameters leads to good performance [42]; therefore, it was determined that comprehensive experimentation with these parameters would add little value and be computationally prohibitive for this application.

We model the fitness of each candidate solution using 5-fold cross-validated area under the receiver operating characteristics curve (AUC, also commonly referred to as the C statistic), which is a standard measure of predictive performance for classification models [43]. We use logistic regression for the classification estimator for two reasons. First, logistic regression is a deterministic algorithm and therefore does not confound the performance of the GA as would a stochastic ensemble approach such as a random forest or boosting algorithm. Second, logistic regression is computationally efficient and therefore allows the GA to explore more generations of candidate solutions for a fixed computation budget.

The specific calculation of fitness depends on the modeling approach for the multilevel data. For the flattening approach, patients only have a positive indication for either a selected complaint or a selected complaint category. Patients with a selected complaint are removed from their corresponding complaint category before the classification model is trained. For example, suppose a complaint for abdominal cramping is selected, which belongs to the more general abdominal pain category. Therefore, patients with the specific abdominal cramping complaint will be removed from the more general abdominal pain category. Patients with complaints that are not selected (e.g., abdominal mass in Figure 1) retain positive indications for the corresponding complaint category (i.e., abdominal pain for this example). This structure maintains a single, mutually exclusive, level for the chief complaint predictor. By contrast, the hierarchical approach retains positive indications for the corresponding complaint category regardless of whether a specific complaint is selected or not. For example, patients with abdominal cramping will have positive indications for both the specific complaint and the corresponding complaint category (abdominal pain). Once the chief complaint specification has been updated for each candidate solution (based on the selected complaints), we calculate the 5-fold cross-validated AUC for the critical care outcome using logistic regression as the classification algorithm and the age, gender, arrival mode, complaint categories, and selected complaints as predictors. The top *N* candidate solutions with respect to fitness are retained for the next generation, and the process terminates when it reaches the prespecified number of generations.

### 2.3. Model Evaluation

We run our GA using the flattened and hierarchical fitness functions for each of the five patient populations and compare the performance of the best-found solutions with two baseline models. The first baseline model only includes the specific complaints for the classification model, whereas the second baseline model only uses complaint categories. We utilize DeLong's method to evaluate the statistical differences in fitness function values between our EC approach and the baseline models [44]. In addition, we evaluate the performance of each model for specific subgroups of patients using a bullseye analogy, in order to characterize any performance differences across relevant subsets of the population. We define the inner region as patients who are directly affected because their specific complaint is selected by the GA. The middle region contains patients who are indirectly affected by a change in their complaint category. Although their specific complaint is not selected, the composition of their complaint category is altered because some patients within the complaint category are treated differently. Finally, the outer region contains patients with no direct connection to patients with selected complaints and is only affected by the overall classification model. We also compare differences in the predicted probabilities for each subgroup between the GA and the baseline models.

In addition to overall model performance, we explore the selected and excluded complaints themselves, which can provide valuable insight as to which complaints are meaningful in this specific context. An advantage of an EC approach is that each candidate solution—and particularly the strongest candidate solutions—provides feedback about the importance of specific complaints. We compare the selected complaints between the flattened and hierarchical approaches for a given population, and we also attempt to draw comparisons across the five populations.

## 3. Results

**Figure 2:**
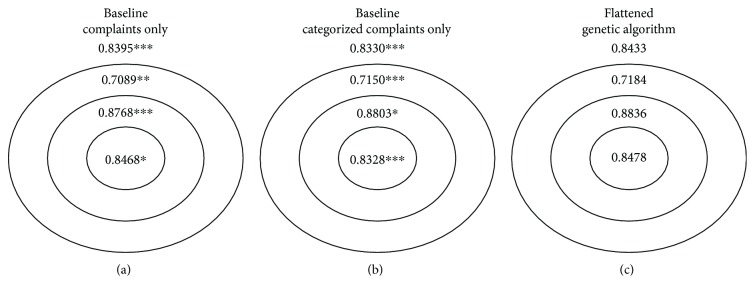
Bullseye performance for baseline models (with specific complaints only and complaint categories only, resp.) and flattened genetic algorithm for the academic hospital. Overall performance is indicated outside of the bullseye. Statistical significance for the difference in 5-fold cross-validated AUC (using DeLong's method) between the flattened genetic algorithm approach and the corresponding baseline models is indicated by ∗∗∗ for *p* < 0.001, ∗∗ for *p* < 0.01, and ∗ for *p* < 0.05.

**Figure 3:**
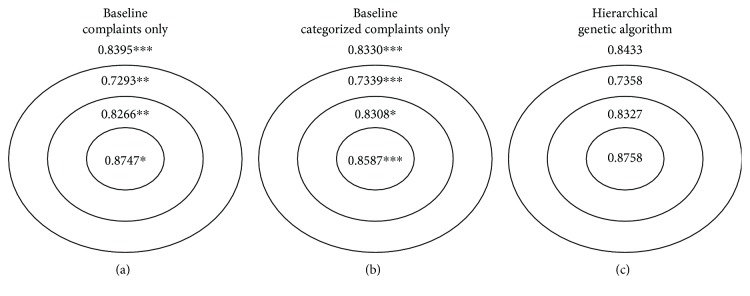
Bullseye performance for baseline models (with specific complaints only and complaint categories only, resp.) and hierarchical genetic algorithm for the academic hospital. Overall performance is indicated outside of the bullseye. Statistical significance for the difference in 5-fold cross-validated AUC (using DeLong's method) between the flattened genetic algorithm approach and the corresponding baseline models is indicated by ∗∗∗ for *p* < 0.001, ∗∗ for *p* < 0.01, and ∗ for *p* < 0.05.

We first present detailed results for the academic hospital and then summarize the results for the other ED populations. In Figures 2 and 3, we summarize the bullseye performance for the flattened and hierarchical approaches, respectively, relative to the two baseline models. We note here that separate figures are required for the comparison due to the distinct selection of complaints by each approach and therefore distinct specifications of the inner, middle, and outer subpopulations.

Overall, both GA approaches demonstrate a statistically significant improvement in the overall 5-fold cross-validated AUC relative to the baseline models (*p* < 0.001 for the both cases), so there is a benefit to including both specific and categorized complaint information for this application. In addition, statistically significant improvements were observed for all subgroups relative to the baseline model with complaints only and for the inner and middle subgroups relative to the baseline model with categorized complaints only. These results suggest that the GAs achieved improvements for multiple subgroups in the population without sacrificing the model's performance on other subgroups.

**Figure 4:**
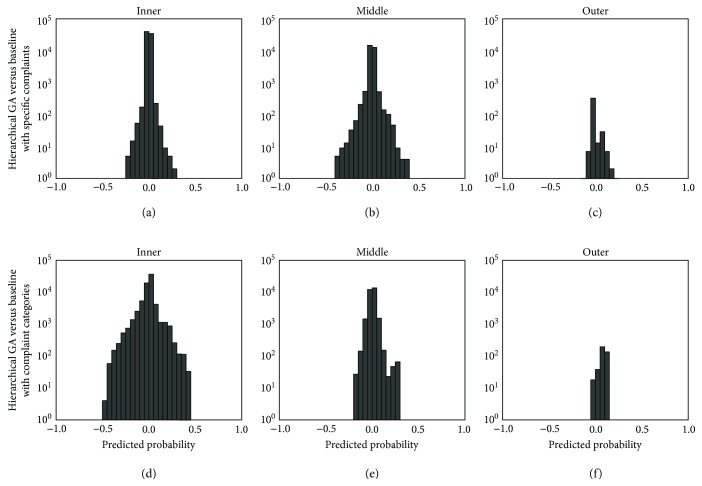
Histogram summary of differences in predicted probabilities of the hierarchical approach relative to baseline models. (a, b, c) Baseline model with complaints only. (d, e, f) Baseline model with categorized complaints only for the inner (a, d), middle (b, e), and outer subgroups (c, f) of patients. Note that the *y*-axis displays the frequency (count) of patients on a logarithmic scale.

In Figure 4, we summarize the differences in predicted probabilities for the hierarchical approach relative to the baseline models. The results for the flattened approach are very similar (not shown). One notable difference is that the predicted probabilities for patients in the inner subgroup are frequently adjusted relative to the baseline model with categorized complaints only. These adjustments are the direct effect of including more specific information (i.e., complaints) in addition to the categorized complaints. Therefore, some patients are shifted toward being a higher risk of a critical care outcome, and others are shifted toward a lower risk, depending on their specific (rather than categorized) complaint. The other notable difference is the significant frequency of adjustments to predicted probabilities for the middle subgroup of patients relative to the baseline model with specific complaint information. The predicted probabilities for these patients are adjusted by augmenting specific complaint information with categorized complaints. Minimal changes are made to the predicted probabilities for the outer subgroups that are not directly affected by the selection of specific complaints.

The performances of the flattened and hierarchical approaches on the four other patient populations were quite similar to their performances on the academic hospital patient population. Specifically, both approaches achieved a statistically significant improvement in overall 5-fold cross-validated AUC relative to the baseline models. In addition, both approaches consistently achieved statistically significant improvements relative to the baseline model with categorized complaints only for the inner bullseye subgroup and relative to the baseline model with complaints only for the middle bullseye subgroups (i.e., the subgroups most directly affected by the EC approach). Statistically significant improvements were observed for other bullseye subgroups, but these improvements were not consistent across all populations. Finally, there were more adjustments for predicted probabilities relative to the baseline model with categorized complaints only than the baseline model with complaints only, particularly for the inner bullseye subgroup.

Overall, there is minimal difference between the performance of the flattened and hierarchical approaches, and there is no significant difference across any of the five populations (see Table 5). In addition, there are similar effects on predicted probabilities (as in Figure 4), in that the most substantial effects are differences for the inner subgroup relative to the baseline model with categorized complaints only and for the middle subgroup relative to the baseline model with complaints only.

Despite the similarities in performance, there are some key differences between the two approaches. Training times are much faster for the hierarchical approach (see Table 5), as there is no restructuring of the multilevel data as for the flattened approach. On the other hand, the flattened approach has the advantage of reducing the dimensionality of the data into a single level, which could improve run times once the multilevel structure has been reduced into a flattened format. We note, however, that prediction times using either trained model would be very fast. Finally, there is significant disagreement among the selected complaints for a given population (see Table 5). In general, the two approaches only agree on approximately 55–60% of the complaints to either exclude or select in the predictive model for critical outcomes for ED patients. The remaining complaints were uniquely selected by only one approach.

## 4. Discussion

This EC approach demonstrates a statistically significant improvement over single-level models that use only complaint or categorized complaint information. These improvements are significant not only for the overall population, but for directly affected subgroups within the population without sacrificing performance on others. It is important to note that these improvements, although seemingly small in magnitude, would have a significant impact over the large volume of patients who visit the ED. Similar (in magnitude) improvements were observed in previous work relative to their selected baseline models [29, 30], although their performance was only evaluated at the overall (not the subgroup) level.

In addition to the performance improvements, this approach reduces the dimensionality of multilevel features. For the flattened approach, multilevel data is collapsed into a single mutually exclusive level. This is advantageous when population size may limit the number of predictor variables that can be meaningfully included. For the hierarchical approach, excluded complaints are pruned from the multilevel data structure. Once enforced, these reductions in dimensionality can facilitate faster development of prediction models, including algorithm selection, parameter tuning, cross-validation, testing, and prediction. The output from these feature selection approaches also provides practitioners with important feedback about the relevance of specific information in the context of a particular outcome. We believe that feature selection—as opposed to using an attention mechanism—has advantages in interpretation over previous approaches.

It is unclear whether the uniquely selected complaints are meaningful in the context of a specific complaint structure (i.e., flattened or hierarchical), or if they are simply insignificant artifacts of the stochastic GA. However, jointly selected complaints have strong support that they are meaningful for a particular outcome, and jointly unselected complaints have strong support that they are not meaningful. Potential improvements to this approach may involve a hybrid solution that leverages output from both approaches. For example, select complaints for the prediction model only if they are jointly selected by both approaches. Or alternatively, select complaints for the prediction model only if they are selected by at least one approach and they meet some minimum sample size requirement.

The stochastic nature of the evolutionary approach may raise questions about its reliability. However, the top candidate solutions for a given run consistently select the same complaints to exclude or include in the prediction model. Very few complaints (<10% for each population) are inconsistently excluded or selected in the prediction model, and for many of these cases, the complaints lean strongly toward being excluded or included (e.g., bladder pain was included in 19 of 20 of the top candidate solutions for the academic hospital).

## 5. Conclusion

In this study, we propose an EC framework for the specification of multilevel data for predictive models. This framework is easy to implement, leverages readily available open-source software, and can be adapted to optimize specification of multilevel data for many predictive applications. This includes the flexibility to accommodate other evolutionary algorithms (e.g., random mutation hill climbing and simulated annealing). The representation of candidate solutions (i.e., binary bit strings) would most likely be similar, and selection, crossover, and mutation operations (and associated control parameters) can be adjusted according to performance. Further, alternative fitness functions may be applied in place of the 5-fold cross-validated AUC. For example, a different cross-validation scheme (e.g., train-test split and stratified cross validation), estimator (e.g., classification tree and regression estimator), or performance measure (e.g., classification accuracy, *R*^2^) could readily be substituted into the framework. In addition, alternate types of preprocessing—similar to the dynamic restructuring of multilevel data for the flattening approach—can be inserted prior to the computation of the fitness function, which is a noted advantage over other feature selection approaches.

We focus here on the specific application of specifying complaint information for predicting critical outcomes for ED patients; however, this approach is generalizable to many types of multilevel data within healthcare. For example, the other key component of the electronic triage algorithm requires prediction of admission outcomes for ED patients. We have applied this framework to this prediction problem as well, and the results are quite similar to those reported here for the critical care outcome.

## Figures and Tables

**Table 1:** Common multilevel predictor data available in electronic health records.

Multilevel predictors	Description	Examples
Reasons for visit	Descriptors of the reason for the healthcare system encounter	Ambulatory care chief complaints; inpatient admission diagnoses
Diagnoses	Descriptors of patients' differential or final diagnosis departing the healthcare system	International classification of disease codes (e.g., ICD-10); read codes
Medical history	Descriptors of previous medical history and chronic conditions	EHR problem lists (e.g., diabetes, previous coronary artery bypass graft (CABG), hypertension)
Diagnostic and therapeutic procedures	Descriptors of diagnostic and therapeutic courses of action taken	Procedure coding system (ICD-10-PCS), surgical procedures, rehabilitation
Diagnostic exams	Descriptors of medical tests conducted	Laboratory exams, imaging exams, physical exams
Medication	Descriptors of medications administered	US Food and Drug Administration Drug Class (e.g., opioids and hydrocodone)
Administrative	Descriptor of the administrative status of patients	Inpatient, outpatient, observation

**Table 2:** Summary of categorical predictor variables (abnormal ranges indicated in bold).

Predictor	Categories	Ranges/categories
Age	8	18–29, 30–39, 40–49, 50–59, 60–69, 70–79, 80–89, >90
Gender	2	Male, female
Arrival mode	2	Via ambulance, walk in
Temperature (°F)^∗^	6	**<94.8**, 94.8–96.1, 96.1–99.2, 99.2–100.4, **>100.4**
Pulse (bpm)^∗^	8	**<49**, 49–59, 59–105, 105–109, 109–119, **119–129**, **>129**
Respiratory rate (bpm)^∗^	6	**<13**, 13-14, 14–19, 19–23, **>23**
Blood pressure (mmHG)^∗^	6	**<99**, 99–106, 106–176, 176–199, **>199**
Oxygen saturation (%)^∗^	4	**<93**, 93-94, >94

^∗^Each vital sign also includes an additional category for missing data.

**Table 3:** Patient population summary.

	ACAD	COMM	BRAZIL	UAE	NAT
Sample size	104.5 K	144.9 K	94.8 K	103.5 K	74.6 K
Unique complaints	686	616	358	288	649
Critical outcome prevalence	3.45%	3.48%	3.00%	1.68%	3.05%

**Table 4:** Summary table of genetic algorithm control parameters and operators.

Parameter	Setting
Population size (*N*)	40
Number of generations	100
Selection	Tournament (*k* = 3)
Crossover operation	Uniform
Crossover rate	0.6
Mixing ratio	0.2
Mutation operation	Bit flip
Mutation rate	0.2
Bit flip rate	0.05

**(a):** 

	Flattened				
	ACAD	COMM	BRAZIL	UAE	NHAMCS
Overall AUC	0.8431	0.8361	0.8261	0.8820	0.8429
Training time (hr)	42.47	78.67	19.89	15.00	29.06
Selected complaints (%)	48.3	52.8	53.4	59.0	49.9

**(b):** 

	Hierarchical				
	ACAD	COMM	BRAZIL	UAE	NHAMCS
Overall AUC	0.8433	0.8364	0.8260	0.8819	0.8436
Training time (hr)	4.93	8.91	3.46	3.27	3.09
Selected complaints (%)	49.3	64.6	55.6	55.6	46.4

**(c):** 

	Comparison				
Difference in overall AUC (*p* value)	0.6144	0.2210	0.7022	0.3622	0.2579
Jointly selected complaints (%)	28.1	33.1	32.4	37.5	27.5
Jointly excluded complaints (%)	30.6	27.6	23.5	22.9	31.2
